# MEDICATION ADHERENCE AMONG ELDERLY PATIENTS RECEIVING HEALTH CARE SERVICES IN A SELECTED HOSPITAL OF WATERBERG DISTRICT, LIMPOPO PROVINCE.

**DOI:** 10.12688/f1000research.175364.1

**Published:** 2026-01-09

**Authors:** T Mavhungu, AG Mudau, TR Lebese

**Affiliations:** 1 Department of Public Health, University of Venda, Thohoyandou, Limpopo, South Africa

**Keywords:** Keywords: chronic diseases, elderly, Medication adherence

## Abstract

**Introduction:**

Medication adherence among elderly patients is crucial for maintaining their health and well-being; many face challenges that hinder their ability to follow prescribed regimens. This study aims to explore medication adherence among the elderly. The findings aim to make a significant contribution to improving the adherence rate among elderly patients **.**

**Methods and analysis:**

Data will be collected through interviews with two central questions, and probing will be used based on the participants’ responses. The collected data will be analysed thematically.

**Ethics and dissemination:**

The proposal has been approved by the Research Ethics Committee of the University of Venda. Permission to conduct the study will be obtained from the Limpopo Department of Health, the Waterberg District, and the selected hospital. Autonomy, confidentiality, care for the vulnerable group, and informed consent will be maintained. Key findings will be disseminated through academic publications, community settings, policymakers, and healthcare providers in a selected hospital.

## Introduction

Medication adherence among elderly patients living with chronic diseases continues to be a significant challenge in health care. Medication adherence refers to the extent to which a patient’s behaviour corresponds with taking medicine optimally, and it is a key factor in achieving therapeutic goals and improving patient outcomes ( Specialist Pharmacy Service, 2023). Medication nonadherence is associated with poorer health outcomes when an individual fails to achieve the expected health benefits from medications related to a specific illness. Several studies have explored medication adherence among elderly patients with chronic diseases living in South Africa ( AbdELwhab et al., 2025; Gumede et al., 2024; Hung et al., 2020). They recommended different strategies to improve medication adherence among the elderly with chronic diseases. Chronic diseases can influence your ability to work and lifestyle; they have an impact on mental, physical, social, and financial aspects, and they may be a burden to the family members who are living with a chronic patient. Therefore, it is crucial to explore medication adherence among elderly patients with chronic disease(s).

The World Health Organisation (2025) estimates that globally, the proportion of people aged 60 years or older will double by 2050. Most older people reside in low- and middle-income countries, such as South Africa. Population ageing is a public health concern that could negatively impact the South African economy and health system if the government fails to prepare for this change ( Sibanda et al., 2021). There is an increasing burden of the elderly population in many countries, with an estimated total of 2.37 billion people aged 65 years or older globally by 2100 ( Hung et al., 2020). The population of South Africa grew by 19,8% between 2011 and 2022. In 2022, the estimated total population of South Africa was 62,027,503 and included more than five million people aged 60 or older, which represents a 9,2% share of the overall South African population. The number of older persons increased across all provinces; Limpopo had a total population of 6,572,721, comprising 6.9% of the older population (65 years and above). The Waterberg district (Modimolle-Mookgophong municipality) had a total population of 130,113, comprising 9.8% of the elderly population ( SA STATS, 2023).

The elderly face unique health challenges compared to young people; for example, noncommunicable diseases such as cardiac diseases, cancer, and diabetes are causes of morbidity among the elderly. The increased prevalence of comorbidities and the severity of diseases in old age will result in a higher demand for healthcare in the future. The decade of healthy ageing (2020-2030) provides an opportunity for a collaborative effort between the government and other stakeholders to enhance the health and well-being of the elderly ( Sibanda et al., 2021). Globally, non-communicable diseases killed at least 43 million people in 2021, equivalent to 75% of non-pandemic-related deaths ( World Health Organisation, 2025). South Africa is experiencing a growing proportion of the elderly population with rapid increases in the burden of non-communicable diseases. In South Africa, a significant portion of the elderly population, approximately 85%, has at least one chronic health condition, and 60% have two or more ( SA STATS, 2023). Rural areas in South Africa represent a smaller fraction of the population; however, they share a relatively higher burden of non-communicable diseases. SA represents one of the most rapidly ageing countries in sub-Saharan Africa.

In South Africa, the burden of chronic diseases is significant, with deaths from cardiovascular disease, cancer, diabetes, and chronic respiratory diseases increasing by 58.7% over 20 years ( SA STATS, 2023). There are deaths due to major non-communicable diseases over 20 years, from 1997 to 2018, which are the following: cardiovascular diseases increased from 54,701 in1997 to 80,133 in 2018; cancer increased from 27,052 in 1997 to 43,613 in 2018; chronic lower respiratory diseases rose from 10,829 in 1997 to 13,579 in 2018, and diabetes increased from 10,846 in 1997 to 26,880 in 2018 (SATS SA, 2023). In 2018, females accounted for 65,845 deaths due to three non-communicable diseases, while males accounted for 54,556; the median age at death in years was 65 for males and 69 for females. KwaZulu-Natal, Gauteng, Western Cape, and Eastern Cape have some of the highest mortality rates due to non-communicable diseases; this may reflect their population, as they host almost two-thirds of South Africa’s population.

About 50 % of adults struggle with medication adherence ( Nguyenal et al., 2024). Taking medication correctly may seem like a personal or straightforward matter. However, nonadherence or not taking medication as prescribed is a complicated and common problem, and people do not realise the actual damage or side effects of non-adherence. There are several reasons why people may be unable to take medication as directed. Medication adherence issues are common among elderly patients, regardless of whether they are treating acute or chronic diseases. The elderly are susceptible to several comorbidities requiring polypharmacy, and it may make them more likely to adhere to medication than younger patients; they are, therefore, likely to have more hospital readmissions, increased health spending, slow healing, etc. ( Varghese et al., 2024). ( Kvarnstrom et al., 2021) classified factors influencing medication adherence among elderly patients as patient-specific barriers, an illness-specific situation, medication-related issues, health care and system-related issues, as well as logical and financial factors.

Several studies have explored medication adherence among elderly patients with chronic diseases and recommended strategies to improve adherence, including medication management, health education, supportive social networks, and ensuring continuity of care ( AbdElwhab et al., 2025; Gumede et al., 2024; Hung et al., 2020). However, there is a lack of context-specific research addressing medication adherence among elderly patients aged 60 years and above with chronic diseases in the Waterberg district, specifically around the Modimolle-Mookgopong municipality. Furthermore, there is a limited understanding of medication adherence and the facilitators affecting medication adherence among the elderly population within the district, which hinders the development of targeted interventions, resulting in a high rate of complications of chronic diseases, a high rate of readmissions, and high mortality due to medication non-adherence among the elderly. Therefore, this study aims to explore medication adherence among elderly patients receiving health care services in a selected hospital in the Waterberg district.

### Objectives

• To investigate the experiences of medication adherence among elderly patients receiving health care services in a selected hospital of the Waterberg district.
• To describe the perception of medication adherence among elderly patients receiving health care services in a selected hospital in the Waterberg district.

## Materials and methods

### Study design

This qualitative study will employ a phenomenological design to explore medication adherence among elderly patients receiving healthcare services in a selected hospital in the Waterberg district. This design enables an in-depth understanding of the experiences and perceptions of medication adherence among elderly patients.

### Population

The target population for this study comprises elderly patients aged 60 years and above residing in the Waterberg District who have been diagnosed with at least one chronic disease and are receiving healthcare services.

### Inclusion criteria

The inclusion criteria for this study are elderly patients aged 60 years or above who have been diagnosed with at least one chronic disease and can provide informed consent.

### Exclusion criteria

The exclusion criteria encompass patients with severe cognitive impairment or mental illness issues that hinder interview participation.

### Sample size

The researcher is anticipating enrolling 15 to 30 participants. The saturation point will determine the final number of participants, ensuring that the diversity and depth of understanding of participants are adequately captured.

### Interview guide

The primary data collection instrument for this qualitative study will be an unstructured interview guide with two central questions, developed to explore the perceptions and experiences of elderly patients with chronic disease(s) regarding medication adherence.

The interview guide will be developed based on an extensive literature review on medication adherence and age-related factors influencing adherence behaviours. The questions will focus on exploring the experiences and describing the perceptions of medication adherence among elderly patients.

Probing will be used to better understand medication adherence among elderly patients. The researcher will ensure that they understand the participant by paraphrasing, encouraging the participant to elaborate more, and listening attentively. The interview guide will be translated into the local language, Sepedi, to ensure that all participants can understand the questions clearly and contribute actively during data collection, thereby minimising language barriers and promoting effective communication.

### Recruitment

The researcher will recruit participants from the outpatient department in the selected hospital. The researcher will greet and conduct introductions to patients in the outpatient department. Explain the purpose of being there and explain the aim, objectives, and target population of the study. Then ask for patients who fit the criteria and are willing to participate. Volunteer participants will be asked to come to a secluded area. A brief session will be done to explain the research objectives, procedures, and ethical considerations. Participants will be asked to sign a consent form, and they will be told that they can withdraw from participating at any time without prejudice. Clarification of any questions or concerns raised by participants will be provided. During data collection, field notes will be documented through written notes. To capture non-verbal expressions, such as gestures, facial expressions, and body language, researchers will take detailed observational notes immediately following interactions. An audio recording will be used to capture an interview, and the recorded data will be transcribed verbatim by the participant.

### Ethical consideration

Ethical approval for this study was obtained from Research Ethics Committee of the University of Venda (clearance number: FHS/25/PH/15/1509).

### Data analysis

Thematic analysis will be used to interpret the qualitative data collected from the interviews. This method enables the identification, analysis, and reporting of themes within the data, providing a rich understanding of the participants’ experiences and perceptions. Qualitative data analysis software such as NVivo may be used to facilitate data coding and organisation, ensuring systematic analysis and traceability of themes.

### Procedures

TRANSCRIPTION

All interviews will be transcribed verbatim to ensure accuracy and facilitate detailed analysis.

FAMILIRAZATION

The researcher will thoroughly read and re-read the transcripts to immerse themselves in the data.

CODING

Initial codes will be generated systematically across the dataset, focusing on meaningful segments related to medication adherence.

THEME DEVELOPMENT

Similar codes will be collected into tentative themes that capture significant aspects of participants’ experiences.

REVIEWING THEMES

Themes will be reviewed and refined to ensure they accurately reflect the data and are distinct.

DEFINING AND NAMING THEMES

Clear definitions will be developed for each theme, and illustrative quotes will be selected to exemplify key findings.

REPORTING

The final themes will be interconnected to provide a comprehensive narrative on medication adherence among the elderly with chronic diseases.

### Dissemination

The researcher will prepare a comprehensive research report to be submitted to a peer-reviewed journal, focusing on public health, the organisation of community meetings, or collaboration with local health communities to communicate findings. The researcher will conduct a presentation session or workshop for healthcare providers at FH Odendaal Hospital and local clinics to share the findings. The researcher will also prepare a policymakers’ brief summarising key findings and recommendations.

## Discussion

Self-reported Medication nonadherence appears to be common in older adults with multimorbidity and polypharmacy. Depression, necessity and concerns should be considered when assessing medication non-adherence in practice ( Felix & Henriques, 2021). ( Horvat et al., 2024) indicated the barriers of medication adherence among the elderly in three categories, which are medication-related barriers where the elderly face several challenges, such as medication instructions, forgetting to renew medication supplies, taking multiple medications, changes in medication regimens, managing the timing and dosage of various drugs, The other category is patient-related barriers, wherein the elderly experience physical difficulties, decline in general fitness and cognitive abilities, health literacy, and common challenges such as vision, hearing, and understanding, The third barrier is related to the healthcare systems or personnel, where the elderly face challenges such as poor communication from healthcare providers. ( Kvarnstrom et al., 2021) also highlighted the barriers to medication adherence and classified them into patient-specific barriers, medication-specific barriers, illness-specific barriers, health and system-specific barriers, social and culture-specific barriers, logical and financial barriers. ( Maffoni et al., 2020) Identified barriers such as patients’ beliefs and concerns about treatment, patients’ beliefs about polypharmacy and prioritisation, patients’ experience and capabilities, prescriber-patient relationship, health literacy, treatment characteristics, and complexity.

### Study status

This study is currently an ongoing process of collecting data. Permission to conduct research from the Limpopo Department of Health, Waterberg district, and the selected hospital has been obtained.

## Data Availability

Data supporting this study will be made available upon request after the research is completed, ensuring adherence to ethical standards. Data will be available from the corresponding author thomphomavhungu4@gmail.com on reasonable request. Access will be granted to researchers who provide clear research purpose and methodology, agree to comply with the University of Venda’s data sharing policies and ethical approval. No data is associated with this article.
