# MEDICATION ADHERENCE AMONG ELDERLY PATIENTS RECEIVING HEALTH CARE SERVICES IN WATERBERG DISTRICT, LIMPOPO PROVINCE: A Qualitative phenomenological study protocol

**DOI:** 10.12688/f1000research.175364.2

**Published:** 2026-04-16

**Authors:** T Mavhungu, AG Mudau, TR Lebese

**Affiliations:** 1 Department of Public Health, University of Venda, Thohoyandou, Limpopo, South Africa

**Keywords:** Keywords: chronic diseases, elderly, Medication adherence

## Abstract

**Introduction:**

Medication adherence among elderly patients is crucial for maintaining their health and well-being; many face challenges that hinder their ability to follow prescribed regimens. This study aims to investigate the experiences to identify barriers and facilitators influencing adherence and to describe the perceptions of medication adherence among the elderly. Health care professionals can tailor targeted intervention to the challenges faced by the elderly to improve medication adherence among elderly patients. Study design this is a qualitative phenomenological study protocol.

**Methods and analysis:**

The target population for this study comprises elderly patients aged 60 years and above residing in the Waterberg District who have been diagnosed with at least one chronic disease and are receiving healthcare services. This study will be conducted in FH Odendaal hospital, in the Waterberg district. The inclusion criteria for this study are elderly patients aged 60 years or above who have been diagnosed with at least one chronic disease and can provide informed consent and have been using medication for at least three months. The researcher anticipates enrolling 15 to 30 participants, stopping when saturation is reached, and no new themes emerge. The data saturation point will determine the final number of participants, ensuring that the diversity and depth of participants’ understanding are adequately captured. The researcher will use purposive sampling. Data will be collected through interviews with two central questions, and probing will be used based on the participants’ responses. The collected data will be analysed thematically.

**Ethics and dissemination:**

The proposal has been approved by the Research Ethics Committee of the University of Venda. Permission to conduct the study will be obtained from the Limpopo Department of Health, the Waterberg District, and the selected hospital. Autonomy, confidentiality, care for the vulnerable group, and informed consent will be maintained. Key findings will be disseminated through academic publications, community settings, policymakers, and healthcare providers in a selected hospital.

## Introduction

Medication adherence among elderly patients living with chronic diseases continues to be a significant challenge in health care. Medication adherence refers to the extent to which a patient’s behaviour aligns with optimal medication use, and it is a key factor in achieving therapeutic goals and improving patient outcomes ( Specialist Pharmacy Service, 2023). Medication nonadherence is associated with poorer health outcomes when an individual fails to achieve the expected health benefits from medications related to a specific illness.

The World Health Organisation (2025) estimates that globally, the proportion of people aged 60 years or older will double by 2050. Most older people reside in low- and middle-income countries, such as South Africa. Population ageing is a public health concern that could negatively impact the South African economy and health system if the government fails to prepare for this change ( Sibanda et al., 2021). The elderly population is growing rapidly in many countries, with an estimated total of 2.37 billion people aged 65 years or older globally by 2100 ( Hung et al., 2020). The population of South Africa grew by 19,8% between 2011 and 2022. In 2022, the estimated total population of South Africa was 62,027,503, including more than 5 million people aged 60 or older, which represented a 9,2% share of the overall South African population. The number of older persons increased across all provinces; in Limpopo, the total population was 6,572,721, comprising 6.9% of the older population (65 years and above). The Waterberg district (Modimolle-Mookgophong municipality) had a total population of 130,113, of whom 9.8% were elderly ( SA STATS, 2023).

In South Africa, approximately 85% of the elderly population has at least one chronic health condition, and 60% have two or more ( SA STATS, 2023). This demographic shift drives a growing chronic burden, raising demand for long-term medication use. However, about 50 % of the elderly struggle with adherence ( Nguyen et al., 2024), and non-adherence contributes to readmissions and mortality, especially where polypharmacy and system constraints intersect. Studies describe patient, drug, and health system factors, but no known study has explored older patients’ perspectives and experiences of medication adherence in the Waterberg district. This study, therefore, aims to investigate medication adherence perspectives and experiences among elderly patients in the Waterberg district.

### Objectives

• To investigate the experiences of medication adherence among elderly patients receiving health care services in a selected hospital of the Waterberg district to identify barriers and facilitators influencing adherence.
• To describe the perception of medication adherence among elderly patients receiving health care services in a selected hospital in the Waterberg district.

## Materials and methods

### Study design

This qualitative study will employ a descriptive phenomenological design to explore medication adherence among elderly patients receiving healthcare services in a selected hospital in the Waterberg district. This design enables an in-depth understanding of the experiences and perceptions of medication adherence among elderly patients. This approach allows for capturing the essence of lived experiences without adding interpretation, aligning with the way they describe their perceptions.

### Population

The target population for this study comprises elderly patients aged 60 years and above residing in the Waterberg District who have been diagnosed with at least one chronic disease and are receiving healthcare services.

### Inclusion criteria

The inclusion criteria for this study are elderly patients aged 60 years or above who have been diagnosed with at least one chronic disease and can provide informed consent and have been using medication for at least three months.

### Exclusion criteria

The exclusion criteria encompass patients with severe cognitive impairment or mental illness issues that hinder interview participation.

### Sampling

Purposive sampling will be used to select participants who meet the inclusion criteria until data saturation is reached, at which point no new themes or insights emerge. The researcher will purposively select participants with varying genders, any chronic disease, a treatment duration of at least 3 months, and any cultural background to capture diverse cultural beliefs.

### Sample size

The researcher anticipates enrolling 15 to 30 participants, stopping when saturation is reached, and no new themes emerge. As suggested by Guest et al. (2006). The data saturation point will determine the final number of participants, ensuring that the diversity and depth of participants’ understanding are adequately captured.

### Interview guide

The primary data collection instrument for this qualitative study will be an unstructured interview guide with two central questions, developed to explore the perceptions and experiences of elderly patients with chronic disease(s) regarding medication adherence.

The interview guide will be developed based on an extensive literature review on medication adherence and age-related factors influencing adherence behaviours. The questions will focus on exploring the experiences and describing the perceptions of medication adherence among elderly patients.

Probing will be used to better understand medication adherence among elderly patients. The researcher will ensure that they understand the participant by paraphrasing, encouraging the participant to elaborate more, and listening attentively. The interview guide will be translated into the local language, Sepedi, to ensure that all participants can understand the questions clearly and contribute actively during data collection, thereby minimising language barriers and promoting effective communication.

Prior to the main data collection, a pretest will be conducted in a private setting at a designated hospital. A small subset of 2 to 3 elderly patients who will not participate in the main study but who meet the inclusion criteria will be recruited. The pretest results will be analysed to determine the clarity, relevance, and general usefulness of the interview guide. Based on the feedback and ideas, changes will be made to improve the interview guide’s questions, wording and flow.

The researcher will conduct the interviews, as they have expertise with the patients as a healthcare professional. To minimise bias and power dynamics, the researcher will engage in reflective journaling to acknowledge and set aside preconceptions, and will use open-ended, neutral questions to encourage participants’ led narratives and create a comfortable, non-judgmental space.

### Recruitment

The researcher will recruit participants from the outpatient department in the selected hospital. The researcher will greet and conduct introductions to patients in the outpatient department. Explain the purpose of being there and explain the aim, objectives, and target population of the study. Then ask for patients who fit the criteria and are willing to participate. Volunteer participants will be asked to come to a secluded area. A brief session will be done to explain the research objectives, procedures, and ethical considerations. Participants will be asked to sign a consent form, and they will be told that they can withdraw from participating at any time without prejudice. Clarification of any questions or concerns raised by participants will be provided. During data collection, field notes will be documented through written notes. To capture non-verbal expressions, such as gestures, facial expressions, and body language, researchers will take detailed observational notes immediately following interactions. An audio recording will be used to capture an interview, and the recorded data will be transcribed verbatim by the participant.

### Ethical consideration

Ethical approval for this study was obtained from Research Ethics Committee of the University of Venda (clearance number: FHS/25/PH/15/1509). Permission to conduct a study was obtained from the Limpopo Department of Health, the Waterberg District, and the management of the selected hospital (FH Odendaal). This study will adhere to ethical guidelines concerning human participants. Informed consent will be obtained, participants’ confidentiality will be maintained by ensuring that all the information collected, including audio recordings and transcripts, remains confidential, data will be stored securely in password protected electronic files and locked physical cabinets, care for vulnerable groups will be considered, and participants’ identities will be protected by assigning unique codes or pseudonyms to all collected data. Participants may withdraw from the study at any time without prejudice.

### Data analysis

Thematic analysis will be used to interpret the qualitative data collected from the interviews. Analysis will follow Brauna & Clarke (2006) six-phase thematic analysis approach. This method enables the identification, analysis, and reporting of themes within the data, providing a rich understanding of the participants’ experiences and perceptions. The primary researcher will perform coding, and the second researcher will independently code a subset of transcripts to enhance dependability. Disagreements in coding or theme interpretation will be solved through discussion and consensus. Qualitative data analysis software such as NVivo may be used to facilitate data coding and organisation, ensuring systematic analysis and traceability of themes. Trustworthiness will be maintained by implementing the following: Credibility by checking on participants throughout the data collection process, Dependability by doing an audit trail to document each step, Confirmability by practising reflexivity through reflective journaling throughout the research process to minimise research bias and Transferability by doing a detailed description of the research context, participants’ characteristics and methodology.

### Procedures

TRANSCRIPTION

All interviews will be transcribed verbatim to ensure accuracy and facilitate detailed analysis.

FAMILIRAZATION

The researcher will thoroughly read and re-read the transcripts to immerse themselves in the data.

CODING

Initial codes will be generated systematically across the dataset, focusing on meaningful segments related to medication adherence.

THEME DEVELOPMENT

Similar codes will be collected into tentative themes that capture significant aspects of participants’ experiences.

REVIEWING THEMES

Themes will be reviewed and refined to ensure they accurately reflect the data and are distinct.

DEFINING AND NAMING THEMES

Clear definitions will be developed for each theme, and illustrative quotes will be selected to exemplify key findings.

REPORTING

The final themes will be interconnected to provide a comprehensive narrative on medication adherence among the elderly with chronic diseases.

### Dissemination

The researcher will prepare a comprehensive research report to be submitted to a peer-reviewed journal, focusing on public health, the organisation of community meetings, or collaboration with local health communities to communicate findings. The researcher will conduct a presentation session or workshop for healthcare providers at FH Odendaal Hospital and local clinics to share the findings. The researcher will also prepare a policymakers’ brief summarising key findings and recommendations.

## Discussion

This study will address the identified gap of the absent of known study in investigating the elderly patients’ own perspectives and experiences of medication adherence in the Waterberg district. The findings are expected to deepen insight into their experiences and perceptions. The results are anticipated to assist elderly patients living with chronic diseases and their families. This study is also anticipated to recommend strategies to policymakers to design targeted interventions and to help the government reduce health care costs associated with hospital readmissions, emergency room visits, and the need for expensive medications due to medication non-adherence.

### Study status

This study is currently an ongoing process of collecting data. Permission to conduct research from the Limpopo Department of Health, Waterberg district, and the selected hospital has been obtained.

## Data Availability

Data supporting this study will be made available upon request after the research is completed, ensuring adherence to ethical standards. Data will be available from the corresponding author thomphomavhungu4@gmail.com on reasonable request. Access will be granted to researchers who provide clear research purpose and methodology, agree to comply with the University of Venda’s data sharing policies and ethical approval. No data is associated with this article.
