# Supplementary material for: MEDICATION ADHERENCE AMONG ELDERLY PATIENTS RECEIVING HEALTH CARE SERVICES IN WATERBERG DISTRICT, LIMPOPO PROVINCE: A Qualitative phenomenological study protocol
Source: F1000Res. 2026 Apr 16;15:40. Originally published 2026 Jan 9. [Version 2] doi: 10.12688/f1000research.175364.2 (PMC13090761; doi:10.12688/f1000research.175364.2)
Supplement: Peer review history

# Reviewer report: Reviewer response for version 2

**DOI:** 10.5256/f1000research.198298.r475699

**Published:** 2026-04-24

**Authors:** Tri Yulianti

**Affiliations:** 1 Universitas Muhammadiyah Surakarta, Surakarta, Central Java, Indonesia

The author has done an excellent job of revising the manuscript, making the research methods much clearer.

Is the study design appropriate for the research question?

Yes

Is the rationale for, and objectives of, the study clearly described?

Partly

Are sufficient details of the methods provided to allow replication by others?

Partly

Are the datasets clearly presented in a useable and accessible format?

Not applicable

Reviewer Expertise:

pharmacy practice-based studies, clinical pharmacy, and medication management.

I confirm that I have read this submission and believe that I have an appropriate level of expertise to confirm that it is of an acceptable scientific standard.

# Reviewer report: Reviewer response for version 2

**DOI:** 10.5256/f1000research.198298.r475700

**Published:** 2026-04-17

**Authors:** Mohammed Alfaqeeh

**Affiliations:** 1 Universitas Padjadjaran, West Java, Indonesia

Thank you for the opportunity to review the revised manuscript. The authors have addressed the previous comments well and have significantly improved the clarity and methodological rigor of the study protocol. The revised version is clearer, more comprehensive, and suitable for indexing in its current form. **No further comments**.

Is the study design appropriate for the research question?

Yes

Is the rationale for, and objectives of, the study clearly described?

Partly

Are sufficient details of the methods provided to allow replication by others?

Partly

Are the datasets clearly presented in a useable and accessible format?

Not applicable

Reviewer Expertise:

Public health researcher trained in epidemiology and health economics, focusing on communicable and non-communicable diseases, vaccine policy, ageing populations, and evidence-based health decision-making. Experienced in systematic evidence synthesis, pharmacoeconomic modelling, and translating research findings into policy recommendations.

I confirm that I have read this submission and believe that I have an appropriate level of expertise to confirm that it is of an acceptable scientific standard.

# Reviewer report: Reviewer response for version 1

**DOI:** 10.5256/f1000research.193343.r452694

**Published:** 2026-04-16

**Authors:** Yosra Alhindi

**Affiliations:** 1 Umm Al-Qura University, Makkah, Saudi Arabia

General Comments:

 This study protocol addresses an important and timely public health issue—medication adherence among elderly patients with chronic diseases. The topic is highly relevant given the increasing burden of multimorbidity and population ageing, particularly in low- and middle-income settings such as South Africa.

 The manuscript is generally well structured, and the use of a qualitative phenomenological design is appropriate for exploring lived experiences and perceptions of medication adherence. However, several aspects of the manuscript require clarification and strengthening to enhance methodological rigor, clarity, and scientific contribution. In its current form, the manuscript is potentially publishable after major revisions.

 Major Comments:

 1. Title:

 The title clearly reflects the topic and population; however, it lacks methodological transparency. Since this is a study protocol employing a qualitative phenomenological design, the study type should be explicitly stated.

 Recommendation:

 Revise the title to include terms such as “A Qualitative Phenomenological Study Protocol.”

 2. Abstract:

 The abstract provides a basic overview but lacks sufficient methodological detail and includes an overstatement of expected outcomes.

 - The study design is not explicitly mentioned.

 - The methods section is too brief.

 - The statement implying improvement in adherence rates is inappropriate for an observational qualitative study.

 Recommendation:

 - Clearly state the study design.

 - Include population, sample size, and data collection methods.

 - Rephrase outcomes to reflect exploration rather than impact.

 3. Introduction:

 The introduction provides relevant background but requires refinement:

 - Some sections are repetitive.

 - The discussion of noncommunicable diseases is overly broad.

 - The research gap is not clearly articulated.

 Recommendation:

 - Reduce redundancy.

 - Focus specifically on medication adherence in elderly populations.

 - Clearly define the knowledge gap.

 4. Objectives:

 The objectives are appropriate but somewhat generic.

 Recommendation:

 - Align objectives with phenomenological inquiry.

 - Include exploration of barriers and facilitators.

 5. Methods:

 a. Study Design:

 Clarify whether the phenomenological approach is descriptive or interpretive and justify its use.

 b. Sampling Strategy:

 Specify the sampling method (e.g., purposive sampling) and explain participant selection.

 c. Inclusion Criteria:

 Include a requirement that participants have been on medication for a minimum duration (e.g., 3 months).

 d. Data Collection:

 Provide more detail on interview domains and whether the interview guide will be pilot-tested.

 e. Data Analysis:

 Specify the analytical framework (e.g., Braun & Clarke thematic analysis) and describe how rigor will be ensured.

 f. Ethical Considerations:

 Clarify how confidentiality, anonymization, and data storage will be managed.

 6. Discussion:

 The discussion section contains repetition and includes findings from previous studies rather than focusing on the expected contribution of the protocol.

 Recommendation:

 - Focus on anticipated contributions.

 - Avoid redundancy with the introduction.

 Conclusion:

 The manuscript addresses a relevant topic with an appropriate qualitative design. However, significant revisions are required to improve clarity, methodological transparency, and overall quality before it can be considered for indexing.

Is the study design appropriate for the research question?

Partly

Is the rationale for, and objectives of, the study clearly described?

Partly

Are sufficient details of the methods provided to allow replication by others?

Partly

Are the datasets clearly presented in a useable and accessible format?

Partly

Reviewer Expertise:

pharmacogy

I confirm that I have read this submission and believe that I have an appropriate level of expertise to confirm that it is of an acceptable scientific standard, however I have significant reservations, as outlined above.

# Reviewer report: Reviewer response for version 1

**DOI:** 10.5256/f1000research.193343.r461351

**Published:** 2026-03-12

**Authors:** Tri Yulianti

**Affiliations:** 1 Universitas Muhammadiyah Surakarta, Surakarta, Central Java, Indonesia

The research to be conducted is very interesting regarding medication adherence among elderly people in the Waterberg region. However, several things need to be considered to improve the clarity of the research to be conducted.

 In the title section, to differentiate this article from published articles from research results, specific research methods can be included, such as protocols for qualitative or phenomenological research.

**Abstract:**

 The last sentence in the introduction states that the study's findings can improve adherence rates, which seems inappropriate given that it is an observational study. The contribution that can be made from the results of this study can be conveyed; e.g., health workers can tailor services to the problems faced by elderly patients or develop interventions to improve medication adherence.

 The objectives in the abstract can be adjusted to align with the manuscript, not only to explore medication adherence in the elderly but also to examine the experiences and perceptions of medication adherence among elderly patients.

 In the methods section, the research design to be used can be explained. Information about the population to be studied, sample size, inclusion criteria, and data collection methods can be added to improve clarity for readers.

**Article:**

 In the introduction section, the first and fifth paragraphs repeat information on existing research on medication adherence in Africa. Such repetition of information should be avoided. It would be better to present the results of previous research and its limitations, as well as information that has not yet been obtained regarding medication adherence among older adults in Africa, thereby highlighting the importance of this research. Then, the fourth and fifth paragraphs contain information that is more relevant to the general population. Since this is an article for geriatric patients, it could be made more specific to their conditions.

 In the methods section, for inclusion criteria, note that this study focuses on medication adherence; therefore, patients must have been regularly using the medication for at least 3 months to ensure they are indeed those who use it. Also, for the types of chronic diseases studied, the diseases can be specified more precisely.

 For the sample size information, it is necessary to explain when the data can reach saturation. Please state whether sampling techniques were used in this study, e.g., purposive sampling.

 In the interview guide, it can be explained which domains or aspects will be studied with patients, e.g., medication knowledge, barriers and facilitators, patient beliefs, and medication-taking habits.

 It is necessary to explain who will conduct the interviews and whether they are competent to do the interviews, because the depth of the data collected depends on the interviewers.

 In the discussion section, the author discusses medication adherence in relation to various factors identified in previous studies, many of which are not entirely relevant. The author could have presented the expected results of the study and the future contributions to be made.

Is the study design appropriate for the research question?

Yes

Is the rationale for, and objectives of, the study clearly described?

Partly

Are sufficient details of the methods provided to allow replication by others?

Partly

Are the datasets clearly presented in a useable and accessible format?

Not applicable

Reviewer Expertise:

pharmacy practice-based studies, clinical pharmacy, and medication management.

I confirm that I have read this submission and believe that I have an appropriate level of expertise to confirm that it is of an acceptable scientific standard, however I have significant reservations, as outlined above.

# Reviewer report: Reviewer response for version 1

**DOI:** 10.5256/f1000research.193343.r461352

**Published:** 2026-02-28

**Authors:** Mohammed Alfaqeeh

**Affiliations:** 1 Universitas Padjadjaran, West Java, Indonesia

**Comments to Authors**

***General Comments***

 This study protocol addresses an important and timely public health issue, medication adherence among elderly patients with chronic diseases in the Waterberg District, Limpopo Province. The topic is relevant given the growing burden of multimorbidity and population ageing in South Africa. The protocol is generally well structured, ethically approved, and proposes an appropriate qualitative phenomenological design; however, some areas require clarification to enhance methodological rigor and transparency. In particular, the authors should more clearly specify the phenomenological orientation, justify the sampling strategy, provide greater detail on the interview and translation procedures, and strengthen the description of thematic analysis and strategies to ensure trustworthiness. With moderate revision, this protocol has strong potential to contribute meaningful contextual insights into medication adherence among elderly patients in this setting.

 ----------------------------

***Title***

 The title clearly identifies the topic (medication adherence), target population (elderly patients), and geographic context (Waterberg District, Limpopo Province), which helps readers immediately understand the scope of the study.

 However, as this is a **study protocol using a qualitative phenomenological approach**, the title would benefit from explicitly indicating the study design. Including terms such as *“a qualitative study protocol”* or *“a phenomenological study protocol”* would improve methodological transparency and align with reporting standards for protocols, allowing readers and indexers to immediately distinguish this work from a completed empirical study. Additionally, the phrase “a selected hospital” is somewhat vague. If confidentiality is not required, consider specifying the type of facility (e.g., district hospital) to enhance contextual clarity. If anonymity must be maintained, a brief justification may be helpful within the manuscript.

**Suggested revision:** *Medication Adherence Among Elderly Patients Receiving Healthcare Services in Waterberg District, Limpopo Province: A Qualitative Phenomenological Study Protocol.*

 ----------------------------

***Abstract***

 The abstract gives a clear overview of the topic and intended exploration of medication adherence among elderly patients.

- It should explicitly state the study design (qualitative phenomenological study/protocol) to distinguish it from a completed study.
- The methods description is too brief; consider adding:

- study population (elderly ≥60 years with chronic disease),
- study setting,
- anticipated sample size (15–30 participants),
- sampling approach and use of in-depth interviews.

- The phrase suggesting the study will “improve adherence rate” may overstate the scope; reword to emphasize understanding experiences, barriers, and facilitators rather than implying impact.

----------------------------

***Introduction***

 The introduction provides relevant background on medication adherence and the growing burden of chronic disease among the elderly globally and in South Africa.

- Demographic statistics are detailed; however, they are presented sequentially. Consider synthesizing them to highlight the key message (ageing population → increased chronic disease burden → adherence challenges).
- Some sections are overly broad (e.g., general discussion of noncommunicable diseases). These could be condensed to maintain a stronger focus on medication adherence in the elderly.
- The local context (Waterberg District) is introduced, but the rationale for selecting this specific setting should be strengthened (e.g., service delivery challenges, rural characteristics, or lack of prior qualitative evidence).
- The literature review lists studies but does not clearly identify the knowledge gap. Explicitly state what is not yet understood (e.g., lived experiences, contextual barriers, health-system influences).
- Consider ending the introduction with a clear transition linking the identified gap directly to the study aim.

----------------------------

***Objectives***

 The objectives are relevant to a qualitative inquiry and appropriately focus on exploring experiences and perceptions of medication adherence among elderly patients.

- The objectives could be more aligned with phenomenological research by emphasizing understanding the lived experience of medication adherence rather than using general terms.
- Consider adding a statement indicating the intention to identify barriers and facilitators influencing adherence, if this is part of the analytical focus described elsewhere in the protocol.

----------------------------

***Methods***

**Study Design**

 The use of a qualitative phenomenological design is appropriate for exploring experiences and perceptions of medication adherence among elderly patients.

- However, the protocol does not specify which phenomenological approach is being followed (e.g., descriptive vs. interpretive). Clarifying the philosophical orientation would strengthen methodological rigor.
- Consider briefly explaining *why* phenomenology is the most suitable design for this research question, beyond stating that it allows in-depth understanding.
- It would also be helpful to indicate whether the study follows any qualitative reporting framework (e.g., COREQ or SRQR) to enhance transparency and credibility.

**Population and Eligibility Criteria**

 The target population (elderly patients aged ≥60 years with at least one chronic disease receiving healthcare services) is clearly defined. Inclusion and exclusion criteria are appropriate; excluding individuals with severe cognitive impairment is reasonable for interview-based research.

- However, the sampling strategy is not specified. Clarify whether participants will be selected using purposive, convenience, or maximum variation sampling, and justify the choice.
- Consider explaining how diversity will be ensured (e.g., gender, type of chronic disease, duration of treatment).
- Since recruitment occurs at the outpatient department, discuss whether this may exclude more frail or home-bound elderly individuals and how this might influence transferability.

**Sample Size**

 The proposed sample size of 15–30 participants, guided by data saturation, is generally appropriate for a phenomenological study.

- However, the concept of data saturation should be briefly defined, including how the researcher will determine that saturation has been reached (e.g., no new themes emerging across consecutive interviews).
- Clarify whether recruitment will stop strictly at saturation or within the predefined range (15–30 participants).
- Consider justifying the proposed range by referencing qualitative methodological literature to strengthen rigor.

**Interview Guide and Data Collection**

 The use of unstructured interviews with probing is appropriate for a phenomenological study exploring lived experiences.

- However, stating that the guide includes only “two central questions” is vague. Consider briefly outlining the nature or domains of these questions (e.g., daily medication routines, challenges, support systems).
- Clarify whether the interview guide will be pilot-tested and refined before full data collection.
- The translation of the guide into Sepedi is appropriate; however, explain whether back-translation or validation will be performed to ensure conceptual equivalence.
- Field notes are mentioned; specify how they will be integrated into the analysis.
- Since the researcher may have previously provided care to participants, consider explaining how interviewer bias and power dynamics will be minimized.

**Data Analysis**

**Comment to Authors**

 The use of thematic analysis is appropriate for identifying patterns in participants’ experiences and perceptions.

- The protocol outlines general steps (transcription, familiarisation, coding, theme development), which is helpful; however, it should reference a specific analytical framework (e.g., Braun & Clarke’s approach) to enhance methodological transparency.
- Clarify who will perform the coding and whether more than one researcher will be involved to improve dependability.
- Describe how disagreements in coding or theme interpretation will be resolved.
- Consider explaining strategies to ensure trustworthiness, such as credibility (member checking), dependability (audit trail), confirmability (reflexive notes), and transferability (thick description).

**Ethical Considerations**

**Comment to Authors**

 Ethical approval from the University of Venda Research Ethics Committee is clearly stated, which strengthens the protocol’s ethical compliance.

- The protocol mentions informed consent, confidentiality, and voluntary participation; however, consider briefly explaining:
  - how confidentiality will be maintained (e.g., anonymisation, secure data storage)
  - how audio recordings and transcripts will be protected
  - how participants will be identified using codes rather than names
- It would also be useful to mention whether participants may withdraw their data after the interview if they choose to do so.

----------------------------

***Discussion***

- Much of the content repeats barriers already cited in the introduction. Consider condensing this section to avoid redundancy.
- Since this is a protocol, the discussion should focus more on:

- the anticipated contribution of the study
- how it will address the identified knowledge gap
- and how findings may inform practice or policy in the local context

- Avoid presenting literature findings in a way that appears to pre-empt or predict your study results.
- If retained, this section could instead be reframed as “Expected Contribution” or integrated into the introduction.

Is the study design appropriate for the research question?

Yes

Is the rationale for, and objectives of, the study clearly described?

Partly

Are sufficient details of the methods provided to allow replication by others?

Partly

Are the datasets clearly presented in a useable and accessible format?

Not applicable

Reviewer Expertise:

Public health researcher trained in epidemiology and health economics, focusing on communicable and non-communicable diseases, vaccine policy, ageing populations, and evidence-based health decision-making. Experienced in systematic evidence synthesis, pharmacoeconomic modelling, and translating research findings into policy recommendations.

I confirm that I have read this submission and believe that I have an appropriate level of expertise to confirm that it is of an acceptable scientific standard, however I have significant reservations, as outlined above.
